# Precise Correlation of Contact Area and Forces in the Unstable Friction between a Rough Fluoroelastomer Surface and Borosilicate Glass

**DOI:** 10.3390/ma13204615

**Published:** 2020-10-16

**Authors:** Chao Wang, Shabnam Z. Bonyadi, Florian Grün, Gerald Pinter, Andreas Hausberger, Alison C. Dunn

**Affiliations:** 1 Polymer Competence Center Leoben GmbH, Roseggerstraße 12, 8700 Leoben, Austria; chao.wang.v@googlemail.com (C.W.); andreas.hausberger@pccl.at (A.H.); 2 Department of Mechanical Science & Engineering, University of Illinois at Urbana-Champaign, 1206 W. Green St. MC 244, Urbana, IL 61801, USA; bonyadi2@illinois.edu; 3 Chair of Mechanical Engineering, Montanuniversität Leoben, Otto Glöckel-Straße 2, 8700 Leoben, Austria; florian.gruen@unileoben.ac.at; 4 Chair of Materials Science and Testing of Plastics, Montanuniversität Leoben, Franz-Josef-Straße 18, 8700 Leoben, Austria; Gerald.Pinter@unileoben.ac.at

**Keywords:** elastomer stick-slip, in-situ microtribometry, machined seals

## Abstract

Stick-slip friction of elastomers arises due to adhesion, high local strains, surface features, and viscous dissipation. In situ techniques connecting the real contact area to interfacial forces can reveal the contact evolution of a rough elastomer surface leading up to gross slip, as well as provide high-resolution dynamic contact areas for improving current slip models. Samples with rough surfaces were produced by the same manufacturing processes as machined seals. In this work, a machined fluoroelastomer (FKM) hemisphere was slid against glass, and the stick-slip behavior was captured optically in situ. The influence of sliding velocity on sliding behavior was studied over a range of speeds from 1 µm/s to 100 µm/s. The real contact area was measured from image sequences thresholded using Otsu’s method. The motion of the pinned region was delineated with a machine learning scheme. The first result is that, within the macroscale sticking, or pinned phase, local pinned and partial slip regions were observed and modeled as a combined contact with contributions to friction by both regions. As a second result, we identified a critical velocity below which the stick-slip motion converted from high frequency with low amplitude to low frequency with high amplitude. This study on the sliding behavior of a viscoelastic machined elastomer demonstrates a multi-technique approach which reveals precise changes in contact area before and during pinning and slip.

## 1. Introduction

Machined seals are also called customized seals, offering specific manufacturing according to the demands of end-users. The customizability, time saving, and cost saving for a small amount enable machined seals to be a better solution than the traditionally molded part. This market is growing fast and will reach a revenue of over USD 2.5 billion by 2021 [1]. Real rough elastomeric surfaces deviate from smooth surfaces in that they can have more edges of contact and retain features like asperities or ridges which will deform more readily than a smooth surface. While ideal sliding is smooth, stick-slip problems can be caused by insufficient lubrication at low speeds and high pressures. Stick-slip motion in seals can degrade performance significantly by causing vibrations that lead to cracks and wear [2]. The mitigation of unstable friction includes increasing movement speed or changing the surface roughness and lubricant. However, the motion profiles are typically fixed, and machining marks are inevitable in reducing finishing operations, which minimizes the cost. Thus, there is a need to understand stick-slip motions between elastomers and hard surfaces, and especially the effects of surface features on contact area evolution during unstable events.

Stick-slip is generally a dynamic cyclic process where an interface driven in shear dissipates energy in a succession of quick slip events separated by phases of pinning [3]. In the pinned phase, the two surfaces stick together, and no gross relative motion occurs. In the slip phase, finite relative motion occurs. Elastomers are particularly susceptible to stick-slip friction due to their compliance and increased surface energy. Under a lateral load, the compliant contacts pin together until internal cohesion reaches its limit, at which time the pinning is released by interfacial slip. The competition between interfacial adhesion and strain energy results in repeated stick and slip events [3,4]. Elastomers are well-suited as seal materials due to the same compliance and high failure strain, as they can sustain large deformations and conform to mating surface features without fracture or yield.

In elastomer-flat contacts, the contact area is a controlling parameter. In compliant contacts, the apparent area of contact can approach the real area of contact because roughness is small compared to the deformation of the elastomer. Ludema and Tabor proposed that the rubber friction can be described with contact area and shear strength [4]. They measured the sliding friction between polymers and hard surface at various speeds and temperatures. They suggested that the contact area and shear strength are low frequency and high frequency processes during the deformation process, respectively. In 1971, Schallamach directly observed the contact area between rubber sliders and a hard counter surface, finding that the contact broke not in single large slip events, but rather local slip waves associated with the tangential compressive stress gradient [5]. For the actuation of a hard surface against an elastomeric seal, we focus on incipient slip and the low-speed conditions. Bartenev et al. [6] found that the contact area remained almost constant at low speed, while at high speeds, the slip speeds are faster than the rates of recoil, so rubber properties play a larger role. In the flipped configuration of a hard hemisphere against an elastomer plate using an optical microscope, Barquins [7] defined the movement as a competition between adhesive dragging and relaxation at low speeds. These studies provide the theoretical background for the extreme case of conformal contact, but their application to rough elastomeric surfaces is limited.

Following the foundational work, Arnolds [8] and Roberts [9] included the consideration of roughness and surface energy on rubber friction when conducting experiments between a roughened rubber and a flat glass [8,9]. For roughened rubber, the friction coefficient increased with speed stably. At high speeds, the mechanisms are better understood, such as the stress relief through Schallamach waves [10]. Above a critical sliding velocity, the behavior transitions from stick-slip motion to steady sliding [11,12,13]. Scaling laws and mean field theories [14] have provided guidance for the slip cascades depending upon the description of the interface as shear-weakening or shear-strengthening. From this, we see an open question as to the specific connections between the contact area of a rough elastomer against glass and the shear forces as slip is initiated. We hypothesize that the contact area changes dynamically under the application of shear forces before slip and during slip, and that modern in situ sliding techniques can capture these dynamic changes. Specifically, based on the theory of junction growth of adhesive asperities [15], we predict that the contact area grows on the application of a shear force. However, there must also be a cascading decrease of contact area for gross slip to occur. Thus, we propose that it is the competition between adhesive surface forces and cohesive forces which not only predicts the speed transition as reported by prior researchers [7,8], but also predicts an increase and decrease in contact area before gross slip occurs.

In this study, we test the hypothesis by systematic slip experiments between a machined elastomeric hemisphere and a glass slide, which are simultaneously observed in bright field microscopy to identify the real area of contact. We mapped the evolving real contact area upon the application of shear and found that both pinned and slipping regions are present. A machine learning strategy was used in the image analysis to accurately follow the lateral position of the pinned region. The influence of sliding velocity on stick-slip behaviors was studied over a range of speeds from 1 µm/s to 100 µm/s. The correlation between real contact area and friction force, together with the movement of the stick region achieved with machine learning, revealed the stick-slip process of a machined seal material against glass. In addition, we identified a critical velocity threshold between “micro” stick-slip and “macro” stick-slip. Finally, the time evolution of the stick-slip was roughly correlated to the creep relaxation of the rubber. This research provides a more comprehensive understanding of the conditions and modes of stick-slip which can occur during the operation of machined seals, and can benefit the design of elastomeric seal surfaces to prevent unstable friction modes, thereby improving the sealing performance.

## 2. Materials and Methods

### 2.1. Materials

**Figure 1:**
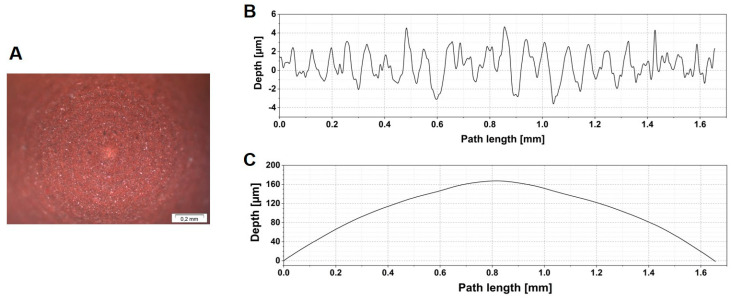
(**A**) Micrograph of FKM hemispherical sample texture (**B**) Roughness and (**C**) waviness of the topography of FKM probe surface.

A fluoroelastomer was selected due to its wide use as a sealing material (FKM, SKF Sealing solutions Austria GmbH, Judenburg, Austria). The test samples were manufactured through a turning process. The whole sample had a cylindrical form with a length of 5.17 mm. One end of the sample was a hemisphere with a diameter of 4.86 mm. FKM has a shore-A hardness of 84. Prior to the experiment, the topography of the sample was characterized with a three-dimensional focus variation microscope (InfiniteFocus, Alicona, Graz, Austria) and an optical light microscope (Stereo Microscope SZX 12, Olympus, Tokyo, Japan). After manufacturing, turning marks were observed on the sample surface (Figure 1A). The surface has an average roughness (Ra) of 1286.0 nm and a mean peak width (RSm) of 104.2 µm. Its waviness (Wa) is 50.2 µm (Figure 1B,C). The Young’s modulus of the glass slide countersurface was over 60 GPa, approximately 4000 times higher than FKM, and because of this, we assumed all deformations occurred in the elastomer specimen. In addition, the countersurface had a roughness less than 2 nm, and as such could be considered smooth in comparison to the probe specimen.

### 2.2. Experimental Procedures

For the creep tests, the normal load was kept constant at 800 mN following loading at 50 mN/s. After reaching the target normal force, it was maintained for ~18 min. Video of the contact spot was recorded for up to 20 min. For the stick-slip tests, the samples were pressed against the glass slide with a normal load of 500 mN and the glass was translated beneath up to a length of 500 µm. Each cycle consisted of two traces in opposite directions, defined here as trace and retrace. In order to investigate the influence of sliding speeds on the stick-slip behavior, four different speeds were selected: 100 µm/s, 20 µm/s, 5 µm/s, and 1 µm/s (Table 1). The number of cycles at each speed was limited to the cycles which showed distinct stick-slip. When this behavior ceased, the test was stopped at the end of that reciprocating cycle.

### 2.3. Microscopy In Situ Microtribometry

The in-situ microtribometer consists of two parts, the tribometer and the optical system. With this setup, it is possible to perform various tests while observing the contact area, e.g., indentation, creep, and friction tests. Prior groups have primarily used one of two experimental set-ups for optical in-situ tests: sliding a hard, transparent hemisphere against a flat specimen [10,16,17], or sliding a hard, transparent flat surface against a hemispherical or flat sample [18,19,20,21,22]. The setup for this work is similar to the latter, in that a 1-mm thick microscope slide is slid under an instrumented probe tip.

**Figure 2:**
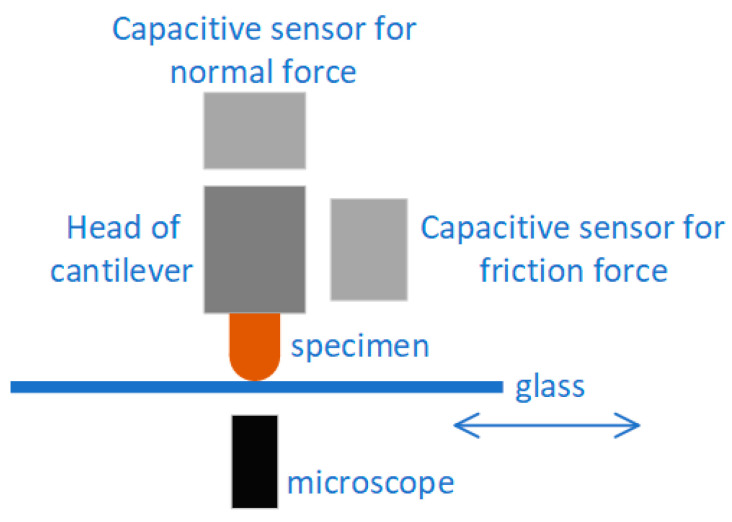
Sketch of the optical in situ microtribometer.

The entire instrument is located atop the stage of an inverted microscope (Figure 2). The test specimen is mounted directly to the end of a cantilevered 4-bar flexure. Two capacitance probes mounted orthogonally to detect micro-motions of the probe in the normal and lateral orientations, which are then translated to forces through the calibrated flexure stiffness. The flexure assembly is translated downward by a linear piezoelectric stage to establish and maintain contact between the test specimen and the glass slide. The glass countersurface was mounted on and reciprocated by a lateral piezoelectric stage of maximum stroke 1.5 mm. The probe approach, load monitoring, lateral motion inputs, and data acquisition were done simultaneously through custom software (LabVIEW, National Instruments, Austin, TX, USA).

The tribometer used in this study has a normal force resolution of about 50 µN, and is regularly used for very low-pressure experiments with soft materials like elastomers and hydrogels [23,24,25]. Thus, the signal-to-noise ratio for the normal load is 500 mN/50 µN, or ~10,000. The 4 × objective was aligned with the probe specimen. Because the motion of the glass slide was controlled independently between the probe specimen and objective, the contact was maintained in the field of view. The sample contact was illuminated through an LED ring illuminator (64 LEDs). Images were acquired using the microscope camera and software at ~20 frames per second (NIS-Elements 4.0 and TI Eclipse. Nikon, Japan).

### 2.4. Image Analysis

Each video was converted to stacked images. Then, each frame was cropped to keep the relevant contact region in the frame. Determining the first frame in which the polymer contacts the glass required removing any external light scattering or noise. To accomplish this, the color values of each pixel of a frame in which we knew the polymer was not in contact with was subtracted from the rest of the frames. As a result, the frame in which first contact occurred became visible. The frames prior to contact were irrelevant and would not be considered in the analysis. The following steps are different for creep tests and sliding tests.

For creep tests, the probe pressed into the glass countersurface at 50 µm/s. The contact point was identified with the method outlined earlier. The image analysis is based on the pixel intensity. Each frame was thresholded using Otsu’s method to compress the greyscale images to binary (black and white) images [26]. After thresholding, the white pixels indicated areas of contact, and conversely, black pixels indicated areas of no contact. To measure the contact area change in time, the area of white pixels was converted to micrometers squared using the dimensions of one pixel, 4.72 µm square.

For sliding stick-slip tests, the subtraction method is not applicable, because the sample deforms with the horizonal movement of the countersurface to some extent. Hence, each frame is directly thresholded using an identical value after cropping.

## 3. Results & Discussion

### 3.1. Creep Test

**Figure 3:**
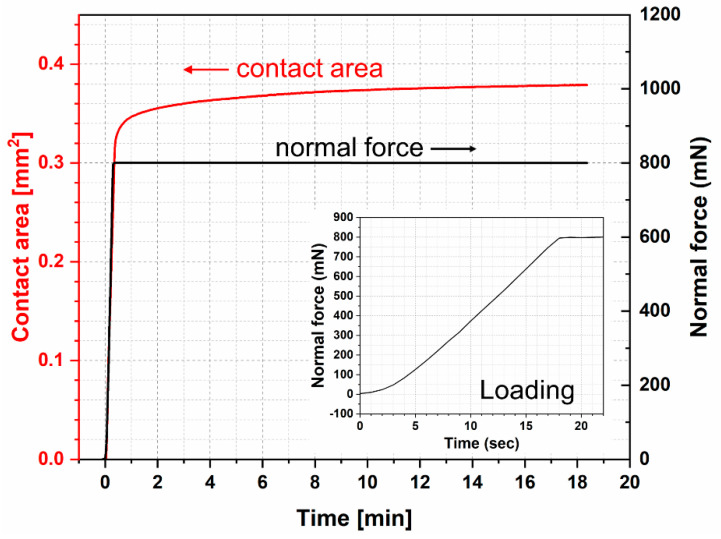
Force-controlled creep test of FKM fluoroelastomer.

Creep is expected to occur in both normal load and friction tests. It is one of the contributions of contact area increase. The contact area was observed from the beginning of the loading phase. As shown in Figure 3, after the loading phase, the contact area increased slowly with time from 0.303 mm^2^ at the end of loading phase and reached 0.378 mm^2^ after 1000 s.

### 3.2. Stick-Slip Friction

#### 3.2.1. General Description of Stick-Slip Behavior

**Figure 4:**
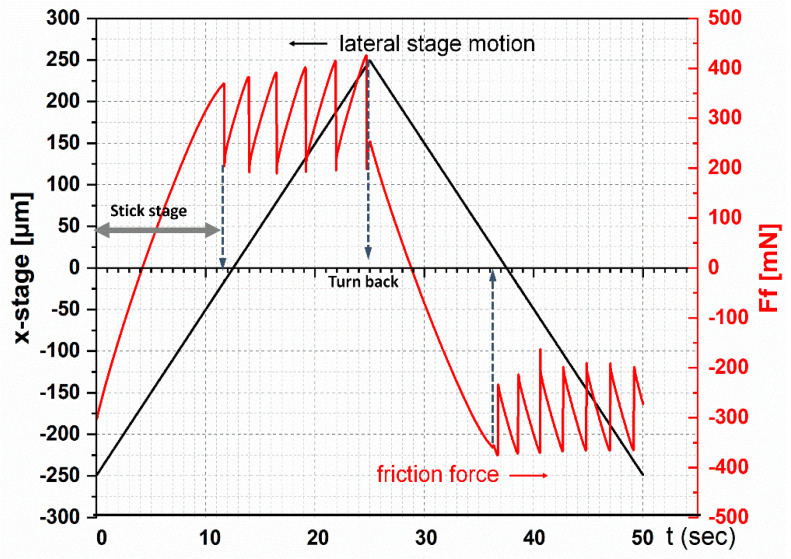
Input displacement of glass countersurface (black) and resulting friction force (red) during the test at a sliding speed of 20 µm/s.

Stick-slip occurred at all speeds tested, but the character changed over time. Due to the relatively low elasticity of FKM and hemispherical geometry of the specimen, slight changes to contact area are detectable. As can be seen in Figure 4, as the countersurface moved from one end of the trace (x = −250 µm) to the other end (x = +250 µm), the frictional force (F_f_) reached a value of 370 mN during the stick stage just before the first slip occurred. At that moment, the countersurface had already moved about 250 µm. In other words, no slip occurred until 250 µm before it reached its deformation limit.

The duration of stick-slip experienced a decay, lasting fewer cycles at lower sliding speeds. For the tests with 100 µm/s or 20 µm/s sliding speeds, the stick-slip behavior is no longer observed after about 20 cycles (20 mm, 200 s for 100 µm/s, 1000 s for 20 µm/s), while it can still be identified after 30 cycles (30 mm, 6000 s) in the 5 µm/s test. For the test with the lowest sliding speed, the stick-slip behavior disappeared after merely four cycles.

#### 3.2.2. Comparison of Stick-Slip Behavior of Different Tests

**Figure 5:**
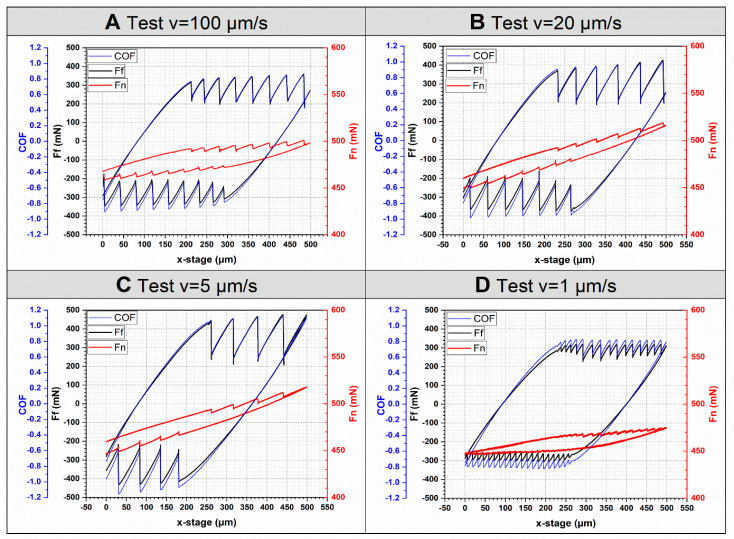
Comparison of the first cycle in four tests. Data shown is the coefficient of friction (blue), friction force (black), and normal force (red From **A** to **D**, the test speed is 100 µm/s, 20 µm/s, 5 µm/s and 1 µm/s, respectively.

The influence of sliding speed can be identified with friction force (F_f_) and the coefficient of friction (COF), in the first cycle in particular (Figure 5). There is a considerable difference between the four test conditions, especially at low speeds. Only focusing on the trace portion, for the tests with decreasing sliding speeds, namely from 100 µm/s to 5 µm/s, eight, six, and four slips took place during the first trace in the tests with 100 µm/s, 20 µm/s, and 5 µm/s, respectively (Figure 5A–C). Therefore, the number of slips reduces with decreasing sliding speeds. However, the test with a sliding speed of 1 µm/s experienced 15 slips in the first trace. At the maximum sliding speed (100 µm/s), a transition from stick-slip to steady sliding was not observed. This result indicates that for an elastomer, there is a critical sliding speed (*V_c_*) above which the stick-slip is “marco” stick-slip. If the speed is lower than *V_c_*, the “macro” stick-slip will become “micro”, which has a smaller amplitude but a higher frequency.

This observation is due to the sliding speed being slow enough to allow the elastomeric material time to recover, resulting in the separation of the adhesive bonds at the interface [27]. Therefore, this critical velocity differs from those defined in other studies [11,12,13] which describe the sliding mode changes from stick-slip to steady sliding. In this study, the critical velocity is found in a low speed regime. The amplitude of stick-slip movement decreases with increasing stick-slip frequency, which is in a good agreement with [28,29,30].

The adhesion force plays an important role in stick-slip sliding tests. Generally, for two flat surfaces in contact, the adhesion force increases with increasing test velocity [31,32]. For smooth surfaces, viscous forces have a greater impact than capillary forces on the adhesion forces at the interface [32,33]. Therefore, higher speeds increase the viscous force, which ultimately increases the adhesion force.

For viscoelastic materials, especially elastomers, the tensile strength increases with increasing strain rates. Thus, when the sliding speed is higher, the material is stiffer. The results indicate that with increasing speed, adhesion force increases slower than shear force. However, at 1 µm/s, the adhesion forces remain low enough to be overcome by the low shear forces, even though the material behaves softer at low speeds. This is in good agreement with the velocity dependence of shear stress reported by another group [34].

#### 3.2.3. Normal Force Response Corresponding to Stick-Slip

**Figure 6:**
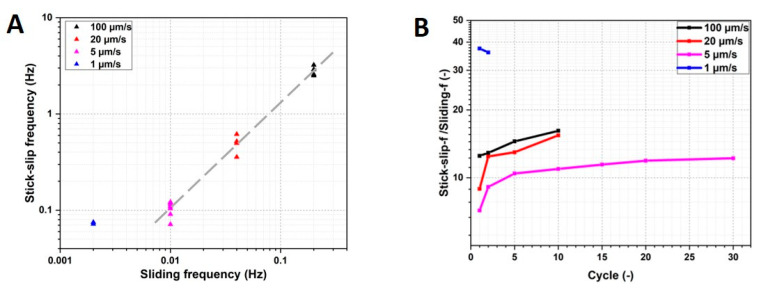
(**A**) Relation of stick-slip frequency and sliding frequency in the first trace. (**B**) Relation of stick-slip frequency/sliding frequency with cycles.

During the stick-slip movement, the penetration depth adjusts to the vibration. Therefore, the normal force (F_n_) changes slightly. This phenomenon is in good agreement with the results from [35]. In terms of reduction of friction force during the slip stage, the largest reduction can be identified in the test with 5 µm/s, which has an average reduction of 245 mN in the first trace. The smallest reduction of friction force occurred in the test with 1 µm/s, with an average value of around 50 mN, which is almost only one fifth of that in the test with 5 µm/s. For the test with 100 µm/s and 20 µm/s, the friction reduction is 130 mN and 210 mN, respectively. As shown in Figure 6, the reduction of friction force increased slightly during the first trace in all tests except the test at 1 µm/s. This phenomenon can be observed in the retrace movement as well.

The first order stick frequency was calculated from the sliding speed and the length of the trace, and there is a trend of more frequent slips at higher speeds (Figure 6A). With the exception of the very low speed regime in which V = 1 µm/s, the trend generally follows a power law fit with an exponent of ~1.2.

The ratio of stick-slip frequency to sliding frequency of the first cycle is close to 10, with the exception of the test at 1 µm/s. With each additional cycle, the ratio increased. The ratios remained nearly the same after the second cycle for the tests at 100 µm/s and 20 µm/s. For the test with 5 µm/s, the ratio stayed nearly constant after five cycles. The behavior of the very low sliding speed test diverged from the trends of the faster tests, and the ratio began at nearly 40 and did not change significantly. The stick-slip behavior was only observed in the first cycle of the test at 1 µm/s.

### 3.3. Real Contact Area Results

**Figure 7:**
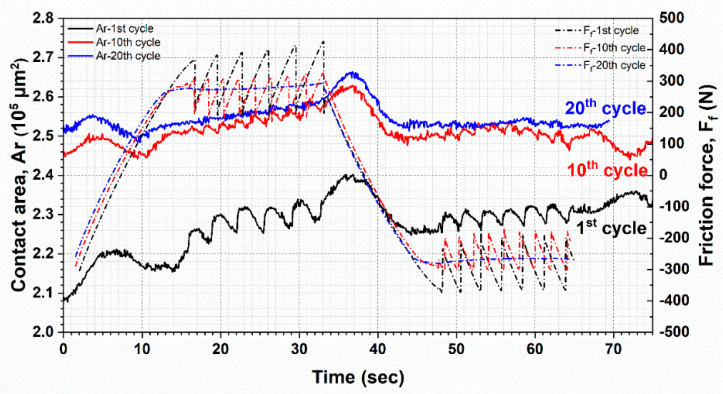
Change of contact area and friction force with time in the first (black), 10th (red), and 20th (blue) cycle from the test at 20 µm/s.

The area in contact also generally showed a frequency response correlated to the stick-slip as measured by forces alone (Figure 7). Within a particular cycle, the contact area reached its maximum value when the glass at the turnaround point between forward and reverse strokes. The magnitude of the contact area was slightly reduced in the reverse stroke, but this corresponded to slightly lower friction forces and may be attributable to slight sample misalignment. As the test proceeded from the 1st to the 10th cycle, the contact area increased, though the change of contact area during the stick-slip behavior was considerably smaller between the 10th and 20th cycles than in the 1st and 10th cycles. This may be attributable to the viscoelasticity of material and the evolving surface of the FKM sample due to creep. As can be seen in Figure 3, the contact area increased by 24.75% after 1000 s with a normal load of 800 mN.

The creep test was fitted using a Burgers model. It is assumed that the half spherical end of specimen is ideal smooth, and the defamation occurs only in the contact area in the same direction as the applied force. The contact follows Hertzian theory. The curve fitting tool in Matlab is used to fit the creep test data. The test data fit excellently with the Burgers model. The fitting was used to estimate the creep effect on the change of contact area during sliding tests. Based on the creep data and Burgers model, the contact area at 500 mN normal force for 1000 s is 0.12675 mm^2^, means an increase of 16.18%, while in the creep test with 800 mN normal force, it increased by 24.75%.

In the sliding test at 500 mN, the contact area increased from 0.10910 mm^2^ to 0.14210 mm^2^ after 1000 s. Hence, the contact area was increased due to creep effect by 0.01765 mm^2^, which contributes to about 53.48% of the total increase. The rest increase can be attributed to sliding movement and subsequently wear. After 20 cycles (1000 s), the specimen slid smoothly over the countersurface as no stick-slip behavior could be identified. This result is slightly counterintuitive in that an increasing contact area with one surface having identical surface energy should result in more adhesion, and therefore a greater propensity to stick rather than a decreased propensity. This may be explained by the changed contact conditions. The contact area of elastomer was turned (Figure 1) and its surface was rough. Hence, due to the interfacial tangential force, wear particles were separated from the bulk material. Small particles facilitated the relative movement. Stick-slip was damped. Between the first and 20th cycles, the average friction force is approximately at the same level and the difference of friction between stick and slip was getting smaller (Figure 7).

#### 3.3.1. Stick Region in the Contact Area

Upon close inspection, the entire real contact area exhibited two regions, a completely pinned, or stuck region, and a slipping region. The pinned central region moves with the countersurface, and the peripheral slipping region has some small relative motion. These regions shift between forward and reverse sliding, and also evolve over the duration of the test. Like the overall contact area, this may be attributed to contact evolution due to plasticity or aging combined with a slight misalignment between the glass surface and the sliding direction.

**Figure 8:**
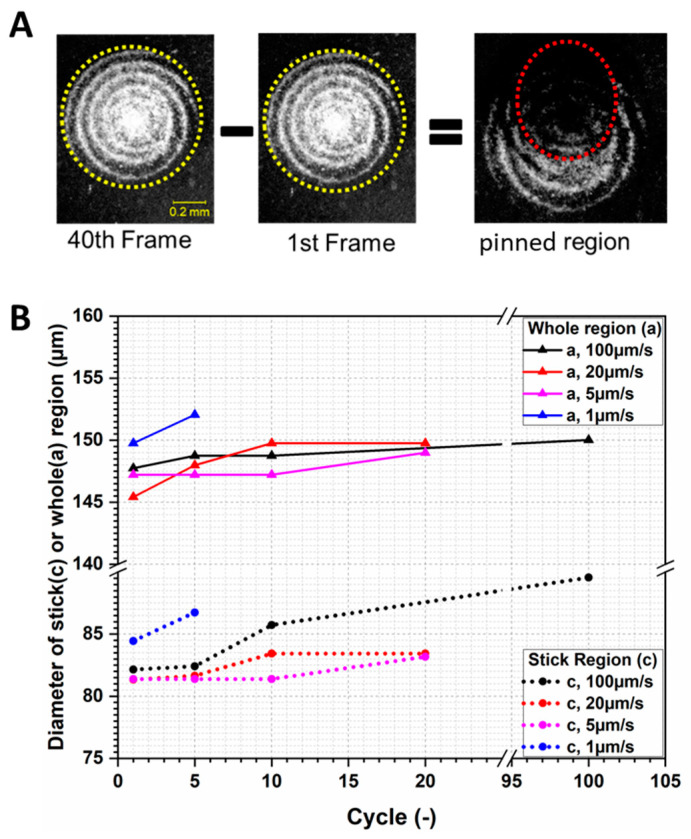
(**A**) The area of the pinned region was calculated by subtracting the 1st frame from the 40th; identical features are assumed to be pinned to the countersurface. (**B**) Change of pinned and slipping regions with cycles.

To calculate the stick region, the countersurface was used as the frame of reference. The pinned region adheres to the countersurface, and they move together. Hence, the pinned region does not experience any relative movement. As can be seen in Figure 8A, the whole contact region was encompassed by the yellow-dashed circles in the 40th and 1st frames. Before being subtracted by the fortieth frame, the first frame was translated according to the prescribed movement of the glass slide. After subtracting the 1st frame from the 40th frame, we identified the region in which the two frames overlap, which remained pinned during the interim. We indicate this region with a red-dashed oval in Figure 8A.

#### 3.3.2. Machine Learning Algorithm for Contact Area Partitioning

The pinned region is a fraction of the whole contact area as shown previously in Figure 8. The complexity of processing this data motivated the use of a machine learning algorithm to understand the movement of the stick region, as exact coordinates of the stick region had to be extracted from the recorded video data. By using the coordinates obtained from manually marking the stick region in about 300 single frames, a convolutional neural network (CNN) was trained to accurately predict the stick region of the remaining 61000 images. The details of the CNN can be found in the Supplementary Material.

These ratios for each cycle of the experiment are shown in Figure 8B. The test at 1 µm/s has the largest contact area, and thus also the largest pinned region of all tested speeds. The creep effect must be taken into consideration. With a 1 µm/s sliding speed, its cycle time is much longer than other tests. Therefore, the viscoelasticity of the material affects the contact area. In addition, generally, for elastomers, the material shows a higher storage modulus with increasing test frequency. For the test with 1 µm/s, the test frequency was 0.002 Hz, which is much lower than the other tests. Meanwhile, its cycle time is much longer than the other tests. Hence, the contact area is slightly larger. For the stick region, the three other tests appear to behave similarly during the first cycle. However, after five cycles, the stick region began to increase at different degrees. As to the whole contact area, only a slight difference can be identified among the three tests.

**Figure 9:**
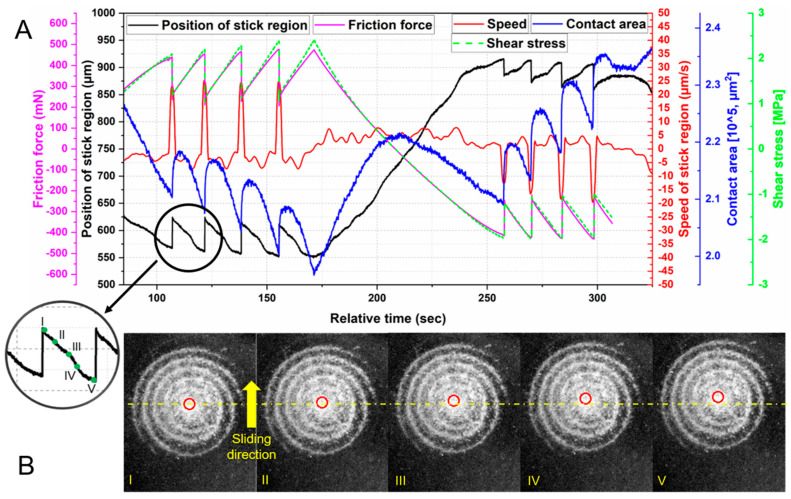
(**A**) Relationship of the movement of stick region, friction force, contact area, and shear stress of the first cycle in the 5 µm/s test; (**B**) Marked stick region within one stick phase (I, II, III, IV, V) using machine learning.

A large composite of the simultaneous stage position pinned region position, friction force, contact area, and shear stress are shown in Figure 9A. The stage position was strongly associated with pinned regions position and friction force. In the stick phase, the pinned region moved with the countersurface at a speed of −5 µm/s. Hence, its position changed proportionally over time. After the stage reversal at t ~ 175 s, the pinned region moved nearly linearly with time, at approximately 5 µm/s.

The movement of the pinned region within one stick phase is shown in Figure 9 and was identified using the CNN. This pinned region moved with the countersurface until a maximum shear stress was achieved. As shown in Figure 9A, the maximum shear stress was reached just before the slip occurred.

#### 3.3.3. Correlation of Friction and Real Contact Area

By observing the contact area during the sliding tests, it is possible to correlate the measured friction force with the contact area, the stick-slip behaviors in particular. The in-situ technique enables a direct correlation of material deformation and the measured forces in real time.

**Figure 10:**
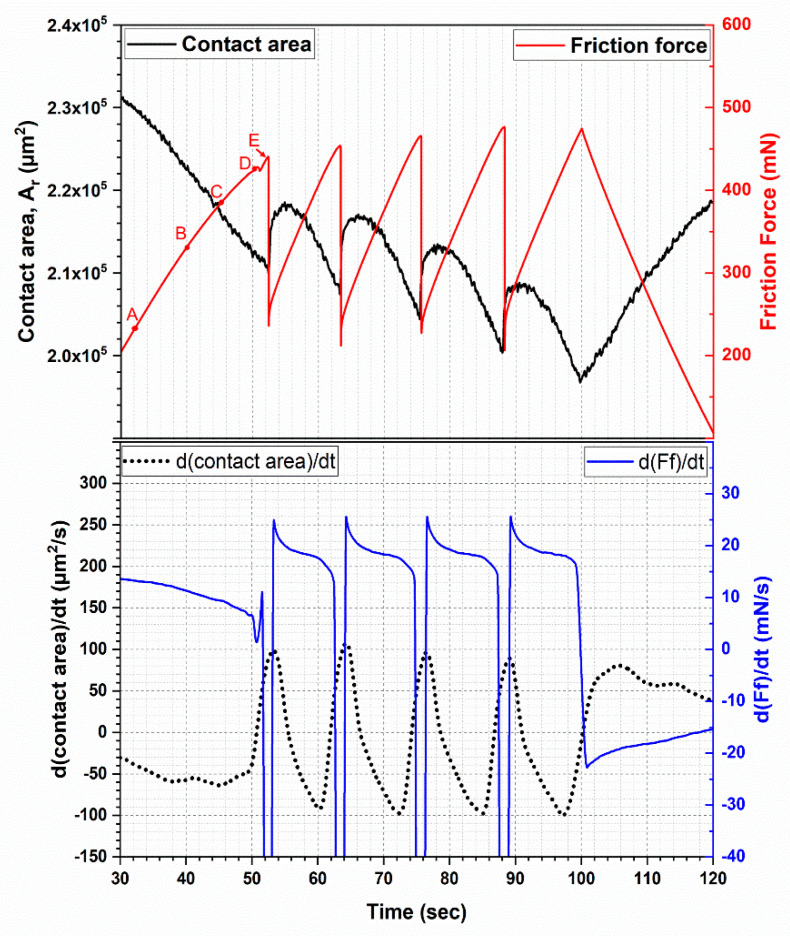
Upper: Change of contact area and friction force within the stick-slip behavior of the first trace in the 5 µm/s test; Lower: Changing rate of contact area and friction force within the stick-slip behavior of the first trace in the 5 µm/s test.

The slope of friction force reduced within a stick phase. As shown in the bottom graph of Figure 10, for all tests, the static friction increased faster at the beginning of the stick phase than at the end. Interestingly, the changing rate of contact area and friction force behave similarly. It is evident that as the stick region becomes smaller, the frictional force increases more slowly. If no slip occurs in the real contact area, the static friction *Q_s_* can be described by the following equation,

(1) $$Q_{s}=\sigma_{g}A_{r}$$

where $\sigma_{g}$ is the shear strength of static contact and *A_r_* is the real contact area. This equation is also used for static friction in [34,36,37,38] and for the sliding tests without stick-slip behaviors [4].

(2) $$\frac{dQ_{s}}{dt}=\underset{Area\;term}{\underset{︸}{\sigma_{g}\frac{dA_{r}}{dt}}}+\underset{Strength\;term}{\underset{︸}{A_{r}\frac{d\sigma_{g}}{dt}}}$$

Thus, the change of static friction can be described with two terms (Equation (2)). The area term takes the rate of change of the real contact area into consideration. As shown in Figure 10, its value decreases throughout the stick phase. However, shortly before slip occurs, it begins to increase, but is still negative. Once slip occurs, the rate of change of the real contact area reaches zero and continues to increase. Therefore, the area term is negative prior to slip. The strength term is strongly impacted by sliding speeds. For thermoplastic materials, the strength increases with increasing testing speeds. Generally, the changing rate of strength decreases with increasing strain. It can be concluded from the changing rate of static friction (Figure 10), that the area term is the dominant term.

However, stick and slip regions can be identified within the contact area during the stick phase. Therefore, the previous assumption is valid at the beginning of the stick phase.

(3) $$Q_{s}=\sigma_{g}A_{stick}+µF_{nslip}$$

(4) $$\frac{dQ_{s}}{dt}=\underset{Stick\;term}{\underset{︸}{\underset{Area\;term}{\underset{︸}{\sigma_{g}\frac{dA_{stick}}{dt}}}+\underset{Strength\;term}{\underset{︸}{A_{stick}\frac{d\sigma_{g}}{dt}}}}}+\underset{Slip\;term}{\underset{︸}{\underset{Force\;term}{\underset{︸}{µ\frac{dF_{nslip}}{dt}}}+\underset{Friction\;term}{\underset{︸}{F_{nslip}\frac{dµ}{dt}}}}}$$

**Figure 11:**
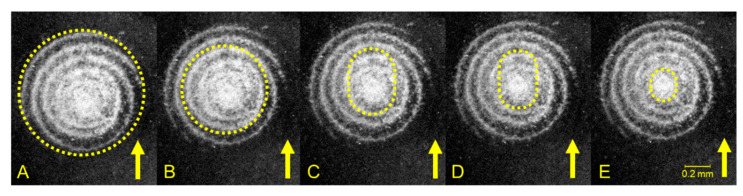
Change of stick area (dotted line) within stick phase of the 5 µm/s test, arrow shows the movement of counter surface; **A**–**E** correspond to Figure 10.

The area of the stick region is *A_stick_* and the normal load of the slip region is *F_nslip_*. The coefficient of friction in the slip region is µ. Regarding the friction force, partial slip part was not mentioned in many studies [4,5,6,7,8,22,39,40]. However, in the partial slip mentioned studies, e.g., [41], the part of slip was not taken into account. As can be seen in Figure 11, the stick area (marked with a dotted line) decreased from A to E, which correspond to the beginning and end of the stick phase, respectively. At the beginning, due to the low elasticity of FKM, the whole contact area (white area in Figure 11) moved with the countersurface. Therefore, no slip took place. With further movement, the tangential force increased with increasing deformation of the sample. Consequently, part of the adhesive bonds began to break, and these areas transformed into the slip region. This process began at the outer ring of the left image of Figure 11, and gradually changed into the stick area illustrated by the ellipse in the right image of the figure. Because part of the contact surface (upper part of marked areas in Figure 11C–E) was a result of the decreasing shear movement, the area term is negative. The slip region became larger from Figure 11A–E, which is why the force term of slip is positive. If µ is assumed to be constant, then the slip term is positive. As a result of decreasing stick area, the area term is the only negative term. Therefore, the static friction depends strongly on the stick area.

The top graph of Figure 10 shows the alternating contact area with the corresponding stick-slip behavior. It can be observed that the contact area decreased during the stick phase and increased abruptly with the slip movement of the specimen. It is understood that the whole contact area deformed largely as a consequence of the shear stress. The outer region of the contact area, the slip region in particular, experiences a transition from the contact state to non-contact state. Prior to the first slip, a small drop of friction force can be identified in Figure 10. This phenomenon has also been identified by other groups [42]. The friction force increased in a nearly linear way until reaching a peak force. At the peak point, the friction force dropped abruptly from over 400 N to around 200 N. At the same time, the static friction transformed into dynamic friction in about 30 milliseconds. Taking a close look at the loading curve, before reaching the peak force, a small drop (about 1%) of friction force during the load phase can be seen. This phenomenon only occurs at low speed regimes (5 µm/s and 1 µm/s). Davis et al. [43] studied the influence of surface wrinkles on the adhesion of elastic materials. They found that, for highly cross-linked, stiffer materials, adhesive forces affect distances shorter than the wrinkle amplitude, which explains why the adhesion behavior is significantly impacted by wrinkles. The separation stress and debonding energy can be nearly doubled by small wrinkles, while large wrinkles show negative effect. Bennewitz et al. [44] found that sliding is preceded by crack-like precursors that cause compressive strain to form along the interface. In addition, the speed of friction force change is shown in Figure 10. In the stick phase, the speed decreased before the friction force reached its peak. In the slip phase, the speed reached over −6000 mN/s (not shown in Figure 10). This precursor phenomenon and the previously mentioned “micro” stick-slip at low speeds can be related to the same mechanisms.

**Figure 12:**
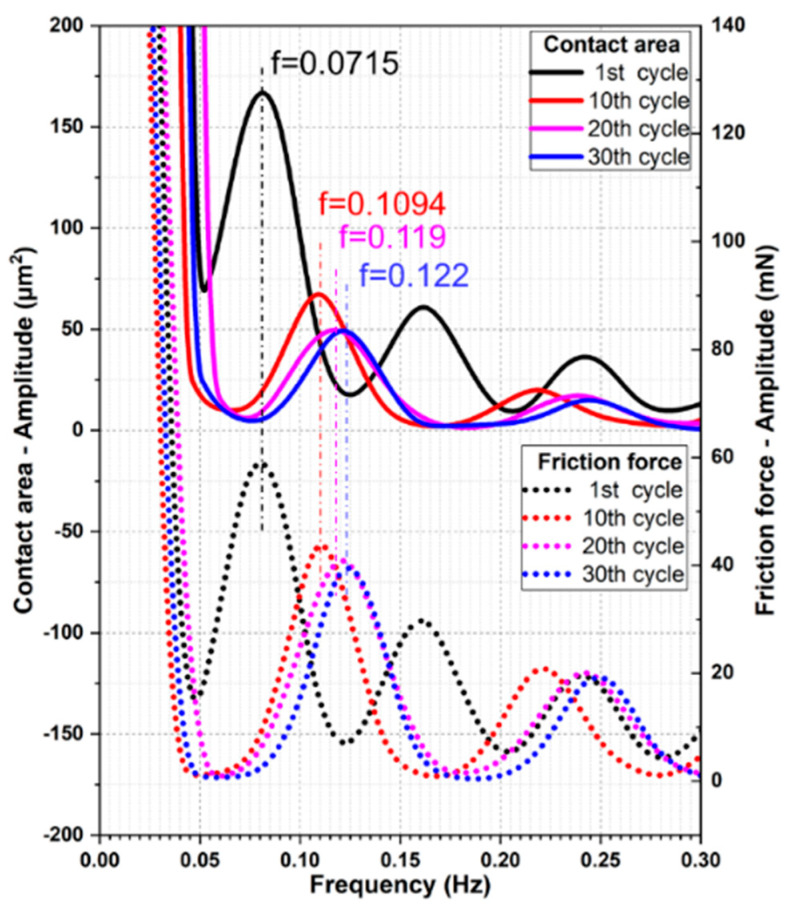
FFT of contact area and friction force of the test with 5 µm/s.

To attain a clear relationship between the alteration of friction force and contact area, the measured friction force and contact area were transformed from the time domain to the frequency domain using fast Fourier transformation (FFT). As shown in Figure 12, the contact area and friction force changed at the same frequency. Additionally, the FFT-frequency increased with cycles, from 0.0715 Hz in the first cycle to 0.122 Hz in the 30th cycle.

## 4. Conclusions

Mechanical seals were investigated in model tests. The elastomeric samples slid on a smooth glass countersurface. The stick-slip behavior was observed and the real contact area was obtained using an in-situ microtribometer. Based on the presented results and discussion, the following conclusions can be drawn

1. The contact area decreased during the stick phase and increased abruptly with the slip movement of the specimen. The transformation from static friction to dynamic friction took about 30 milliseconds.
2. During each macro stick phase, the contact area increased and then decreased with an asymmetric parabolic shape.
3. The shear strength profile was related to loads and speeds using machine learning, which resulted in the discovery of the movement of the pinned region within one stick phase.
4. Two mathematical models of time-resolved friction force and shear stress were introduced and offered an understanding of the phenomena which were observed with a microscope and analyzed from the videos.

Future work could focus on the size change of the stick region and the temperature alteration in the interface during stick-slip sliding.

## Figures and Tables

**Table 1:** Test parameters of stick-slip tests.

Test	Speed [µm/s]	Normal Load [mN]	Length per Trace [µm]	Number of Cycles [-]
1	100	500	500	200
2	20	500	500	20
3	5	500	500	30
4	1	500	500	7

## Supplementary Materials

The following are available online at https://www.mdpi.com/1996-1944/13/20/4615/s1.
