# Supplementary material for: Precise Correlation of Contact Area and Forces in the Unstable Friction between a Rough Fluoroelastomer Surface and Borosilicate Glass
Source: Materials (Basel). 2020 Oct 16;13(20):4615. doi: 10.3390/ma13204615 (PMC7602959; doi:10.3390/ma13204615)
Supplement: Supplementary file 1 [file materials-13-04615-s001.pdf]

materials

SupplementaryMaterials:

Precise Correlation of Contact Area and Forces in the Unstable Friction between A Rough Fluoroelastomer Surface and Borosilicate Glass

Chao Wang [1], Shabnam Z. Bonyadi [2], Florian Grün [3], Gerald Pinter [4], Andreas Hausberger [1] and Alison C. Dunn [2,*]

[1] Polymer Competence Center Leoben GmbH, Roseggerstraße 12, 8700 Leoben, Austria. chao.wang.v@googlemail.com (C.W); andreas.hausberger@pccl.at (A.H)
[2] Department of Mechanical Science & Engineering, University of Illinois at Urbana-Champaign, 1206 W. Green St. MC 244, Urbana, IL 61801, USA. bonyadi2@illinois.edu (S.Z.B)
[3] Chair of Mechanical Engineering, Montanuniversität Leoben, Otto Glöckel-Straße 2, 8700 Leoben, Austria. florian.gruen@unileoben.ac.at (F.G)
[4] Chair of Materials Science and Testing of Plastics, Montanuniversität Leoben, Franz-Josef-Straße 18, 8700 Leoben, Austria. Gerald.Pinter@unileoben.ac.at (G.P)
* Correspondence: acd@illinois.edu Tel: +1 217 300-0349

S1. Details of CNN Training for Contact Area Portioning

The CNN takes as input greyscale images resized to 240 × 240 pixels and outputs a two-dimensional vector corresponding to the relative x- and y- coordinates of the predicted centre of the stick region.

The network consists of six convolutional layers, the first having a kernel size of 5 × 5, the other having a kernel size of 3 × 3. The number of feature maps of the convolutional layers are as follows: 1 (input, greyscale) - 4 - 4 - 8 - 8 - 8 - 4. All convolutional layers are equipped with the ReLu (rectified linear unit) activation function, and the first five convolutional layers are each followed by a 2 × 2 max-pooling layer. After the final (sixth) convolutional layer, there is a fully connected layer of size (196 -> 64) equipped with the ReLu activation, followed by the final fully connected layer of size (64 -> 2) equipped with the Sigmoid activation function.

The network was trained over 45 epochs using the Adam Optimizer, minimizing the mean squared error loss. A batch size of 16 and a learning rate of 0.0005 was used.
